# The spectrum of cardiovascular complications related to immune-checkpoint inhibitor treatment: Including myocarditis and the new entity of non inflammatory left ventricular dysfunction

**DOI:** 10.1186/s40959-022-00147-w

**Published:** 2022-11-24

**Authors:** Maria Sol Andres, Sivatharshini Ramalingam, Stuart D. Rosen, John Baksi, Rajdeep Khattar, Yulia Kirichenko, Kate Young, Nadia Yousaf, Alicia Okines, Robert Huddart, Kevin Harrington, Andrew J.S. Furness, Samra Turajlic, Lisa Pickering, Sanjay Popat, James Larkin, Alexander R. Lyon

**Affiliations:** 1 Cardio-Oncology Service, Royal Brompton Hospital, Guy’s and St. Thomas’ NHS Foundation Trust, Sydney Street, SW3 6NP London, UK; 2 National Heart and Lung Institute, Imperial College London, London, UK; 3 Department of Hospital Therapy N°1, Sechenov University, Moscow, Russia; 4 Royal Marsden Hospital Foundation Trust, London, UK

**Keywords:** Cancer survivorship, Immunotherapy, Myocarditis, Cardiomyopathy

## Abstract

**Background:**

The full range of cardiovascular complications related to the use of Immune checkpoint inhibitors (ICI) is not fully understood. We aim to describe the spectrum of cardiovascular adverse events (cvAEs) by presenting our real-world experience of the diagnosis and management of these complications.

**Methods:**

Two thousand six hundred and forty-seven (2647) patients were started on ICI treatment between 2014 and 2020. Data from 110 patients referred to the cardio-oncology service with a suspected cvAE was collected prospectively and analysed.

**Results:**

Eighty-nine patients (3.4%) were confirmed to have cvAEs while on ICI therapy. Myocarditis was the most frequent event (33/89), followed by tachyarrhythmia (27/89), non-inflammatory left ventricular dysfunction (NILVD) (15/89) and pericarditis (7/89). Results from myocarditis and non-inflammatory left ventricular dysfunction cohorts were compared. Myocarditis and NILVD showed significant differences in respect toof troponin elevation, cardiac magnetic resonance abnormalities and ventricular function. Dual ICI therapy and other immune related adverse events were more frequently associated with myocarditis than NILVD. There was a significant difference in the median time from starting ICI treatment to presentation with myocarditis versus NILVD (12 vs 26 weeks *p* = 0.049). Through early recognition of myocarditis, prompt treatment with steroids and interruption of ICI, there were no cardiovascular in-hospital deaths. NILVD did not require steroid treatment and ICI could be restarted safely.

**Conclusions:**

The full spectrum of cardiovascular complications in patients with immune checkpoint inhibitors is much broader than initially described. Myocarditis remains the most frequent cvAE related to ICI treatment. A novel type of myocardial injury was observed and defined as Atrial tachyarrhythmias and NILVD were also frequent in this cohort. NILVD has a This differs fromdifferent presentation from ICI-related myocarditis, mainly usually presenting afterby the lack of inflammatory features on CMR and biomarkers and a later presentation in time.

## Introduction

Immune checkpoint inhibitors (ICI) are an effective treatment in modern oncology and have become a cornerstone for the treatment of many cancers. ICI activate the immune system to target cancer cells via blockade of co-inhibitory molecules present on T lymphocytes which regulate the immune system. ICI therapy has resulted in a significant reduction in cancer morbidity and mortality in a range of solid tumours, including melanoma, renal cancer, urological malignancies, head and neck cancers, and small cell and non-small cell lung cancer [1]. Activation of the immune system by ICIs is recognised to cause a range of inflammatory immunotoxicities, including myocarditis and pericarditis which have been associated with high morbidity and mortality, as reported in an observational, retrospective, pharmacovigilance study by Salem et al. in 2018 [2]. However, the full range of cardiovascular complications related to the inhibition of immune checkpoints is not fully understood.

Our cardio-oncology service has provided specialist cardiology care to patients from our partner oncology centre, where ICI have been used over the last ten years. Here we describe our real-world experience of diagnosing and managing a range of cardiovascular complications observed in cancer patients receiving ICI therapy.

## Methods

This study is a retrospective analysis of all patients referred to the cardio-oncology service receiving ICI treatment with potential cardiovascular complications. All patients underwent assessment with cardiovascular investigations including a 12-lead electrocardiogram (ECG), measurement of cardiac troponin I, natriuretic peptides (BNP and NTproBNP) and transthoracic echocardiography. Selected patients had cardiac magnetic resonance (CMR) including cine imaging for volumetric analysis, oedema assessment with STIR-T2 imaging and T1 and T2 parametric mapping when available, and late gadolinium enhancement imaging, cardiac positron emission tomography-computed tomography (PET-CT), Holter ECG monitoring, myocardial perfusion scan, CT or invasive coronary angiography, and endomyocardial biopsy, when indicated. Each case was reviewed by the cardio-oncology team and the referring oncology team.

Inclusion criteria for this study were the following:

- Active cancer receiving ICI treatment at the time of referral (ICI treatment was usually interrupted pending the cardio-oncology review).
- New cardiovascular disease without an alternative explanation.

Exclusion criteria were defined as follows:

- Patients who were found not to have any cardiac complications after assessment in the cardio-oncology service.
- Patients who were lost to follow up before completing cardiac investigations.
- Patients referred for assessment of a pre-existing cardiovascular disease who did not develop a new event after starting therapy with immune check-point inhibitors.
- Patients referred for assessment of an intracardiac metastasis to monitor response to ICI therapy.

### Definitions

Immune checkpoint inhibitors related cardiovascular adverse events are defined as all new cardiovascular diseases developed during ICI therapy (within 90 days of a dose of ICI), which are not explained by another cause. The diagnosis of ICI-related myocarditis was based on the definition and classification proposed by Bonaca et al. and classified as possible, probable and definite based on a combination of pathology, imaging, clinical and biomarkers findings [3]. Non-inflammatory left ventricular dysfunction (NILVD) was defined as a new diagnosis of asymptomatic reduction of left ventricular ejection fraction (LVEF) to a value < 50% confirmed by echocardiography or CMR or symptomatic heart failure with LVEF 50–53% with a reduced global longitudinal strain and/or natriuretic peptide elevation. The diagnosis of NILVD requires the exclusion of other causes of acute cardiac dysfunction and the absence of active inflammation on CMR and of a new cardiac troponin elevation.

Immune checkpoint inhibitor-related supraventricular tachyarrhythmias were subdivided into two groups: primary, in which there was no other cardiovascular or systemic ICI-related toxicity, and secondary, where another ICI-related toxicity likely contributed to the risk of the arrhythmia, e.g., thyrotoxicosis, myocarditis, or cytokine release syndrome.

### Statistical analysis

Data were collected prospectively and analysed with the SPSS statistical package (SPSS Inc., Chicago, IL, USA) and R studio Version 1.4.1717. Normally distributed data are presented as mean ± standard deviation. Non-parametric data are presented as median and interquartile ranges (IQR), and categorical data as percentages. Results from myocarditis and non-inflammatory left ventricular dysfunction cohorts were compared using Chi-squared analyses and Fisher’s exact test for categorical variables, two-sided t-tests for continuous variables with Gaussian distribution and Mann-Whitney test for non-parametric variables and a multivariable logistic regression analysis was performed to assess for independent risk factors of myocarditis or NILVD. A *P*‐value of < 0.05 was considered statistically significant.

## Results

**Fig. 1:**
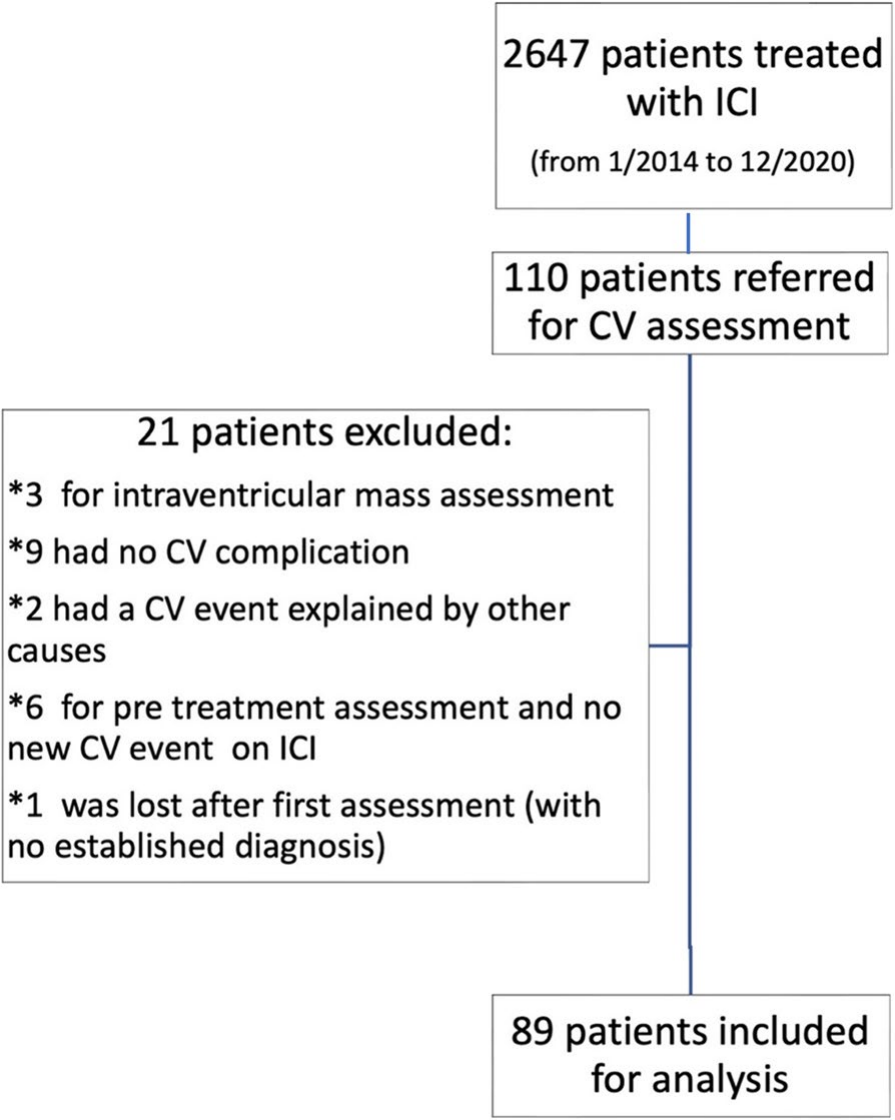
Consort diagram showing patients referred, excluded, and included patients in the study. ICI: immune checkpoint inhibitors. CV: cardiovascular

**Fig. 2:**
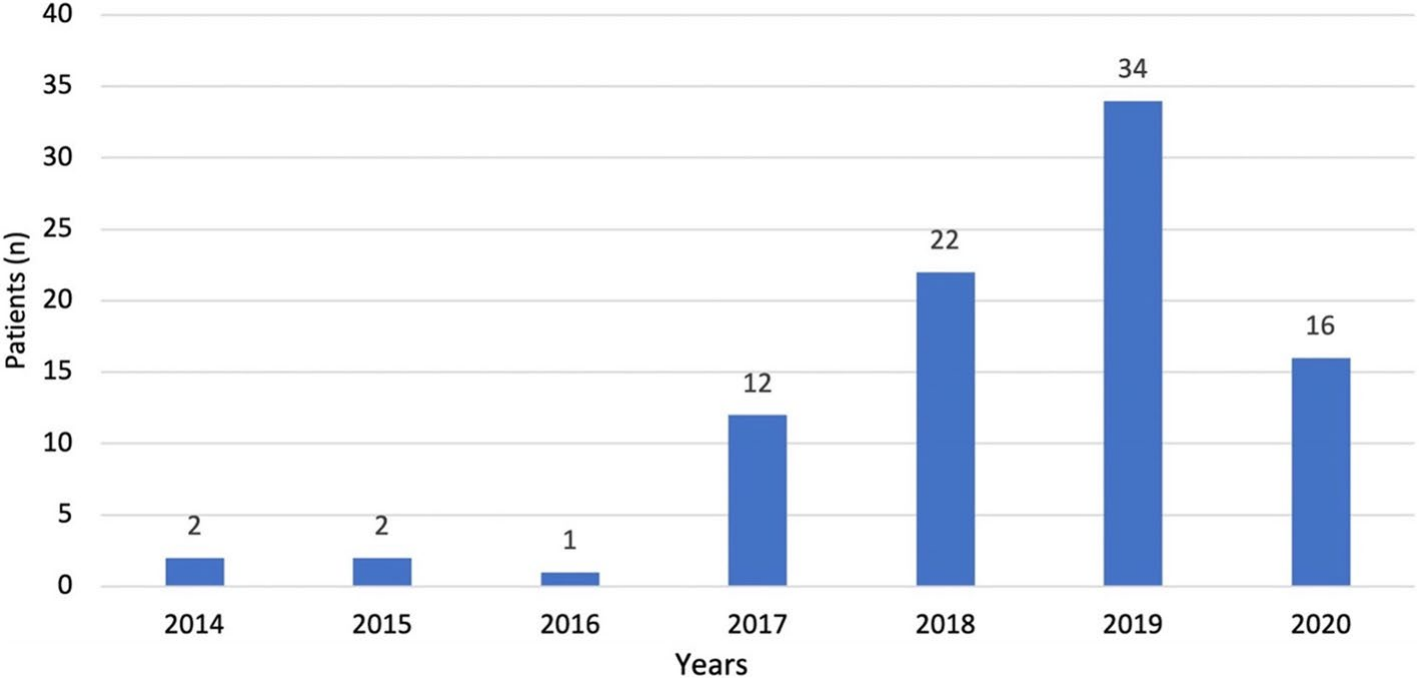
Bar chart showing the number of patients referred per year between 2014–2020. The number of referrals increased steadily over the years since first referral in 2014. In the year 2020, during coronavirus pandemic, oncology and haematology services temporarily paused new immune checkpoint inhibitor prescription. This impacted in the number of referrals to the cardio-oncology service which dropped from 34 to 16 patients

From January 2014 to December 2020, 2647 patients were started on ICI by the oncology and haematology services of our partner oncological centre. Out of this total, 110 were referred to the cardio-oncology service for assessment. Twenty-one patients did not meet the inclusion criteria and were excluded (Fig. 1). Eighty-nine patients were included for the final analysis. There was an increasing rate of referrals from 2014 to 2019, as new indications for immune checkpoint inhibitors were approved, with a mild deceleration in 2020 due to the COVID19 pandemic, which impacted the oncology services to temporarily pause new ICI prescriptions (Fig. 2).

**Fig. 3:**
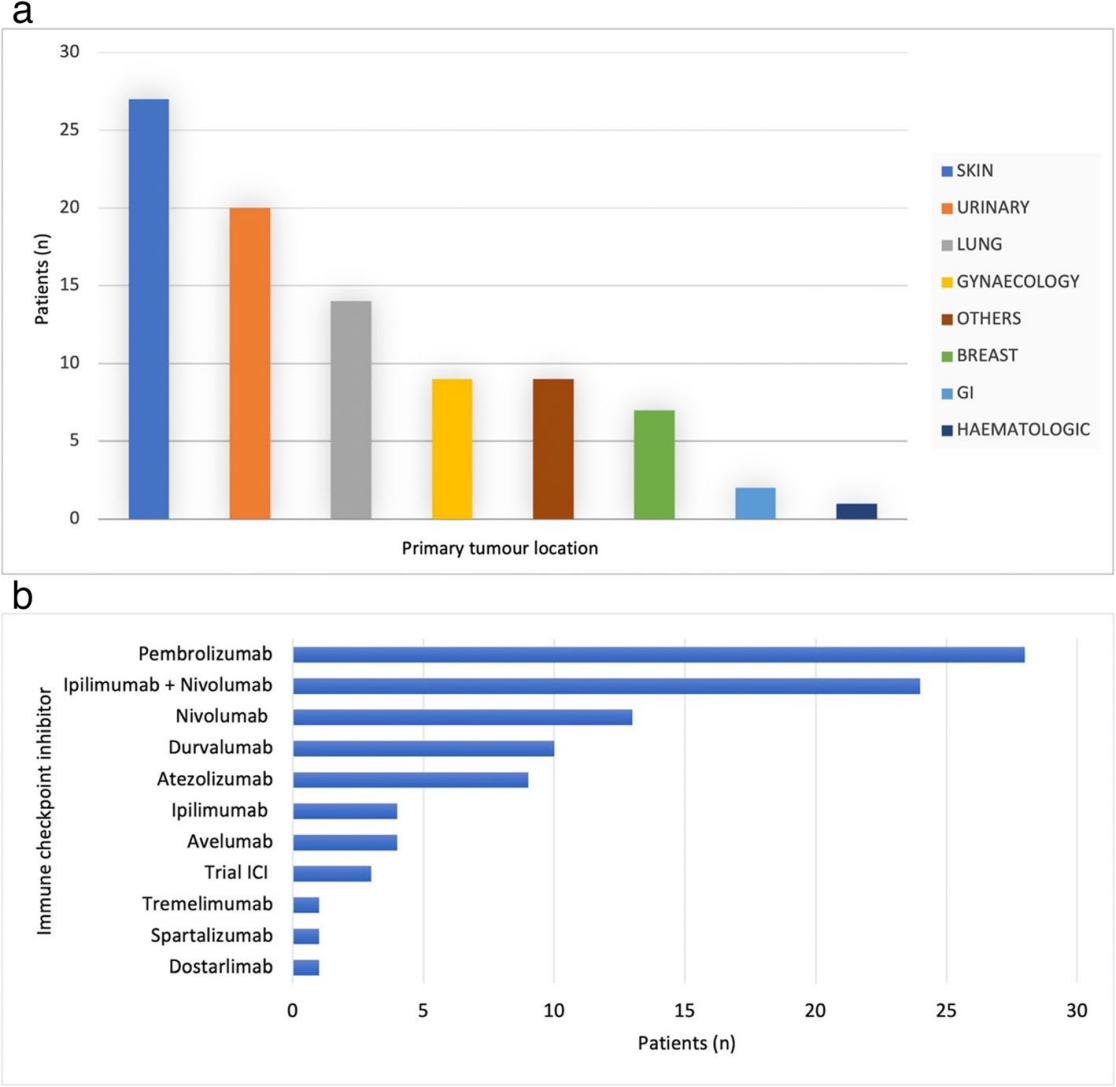
Primary tumour location (**a**) and number of cases with ICI-related cardiovascular toxicity based on the immune checkpoint inhibitor used (**b**). GI: gastrointestinal. ICI: immune checkpoint inhibitor

The demographic characteristics of the population are detailed in Table 1. Median age was 63 years (IQR 51–72) and 55% were men. The most frequent primary tumour type was melanoma, representing 30% of the patients, followed by urinary tract cancer (23%) and lung cancer (16%) (Fig. 3a). The anti PD-1 antibody Pembrolizumab was the most frequently used immune checkpoint inhibitor (28/89), followed by combined therapy (Ipilimumab and Nivolumab) (24/89), Nivolumab as a single agent (13/89), Durvalumab (10/89) and Atezolizumab (9/89) (Fig. 3b).

**Table 1:** Demographic characteristics of the patient included for analysis

Characteristics	*n* = 89
**Age (median, IQR)**	63 (51–72)
**Male (n,%)**	49 (55)
**HTN (n, %)**	25(28)
**DM (n, %)**	6(7)
**Dyslipidaemia (n,%)**	33(37)
**Smoker or Ex smoker (%)**	23 (26)
**Previous cardiac disease (n, %)**	14(16)
**BMI (mean, SD)**	27 (5.5)
**LVEF (median, IQR)**	60 (12)
**LVEF < 50% (n, %)**	17(19)

Previous cardiovascular disease included: valvular disease, coronary artery disease, myocardiopathy, arrhythmias. *HTN *Hypertension, *DM *Diabetes mellitus, *BMI *Body mass index,  *LVEF*Left ventricular eject fraction

### Cardiovascular adverse events

**Fig. 4:**
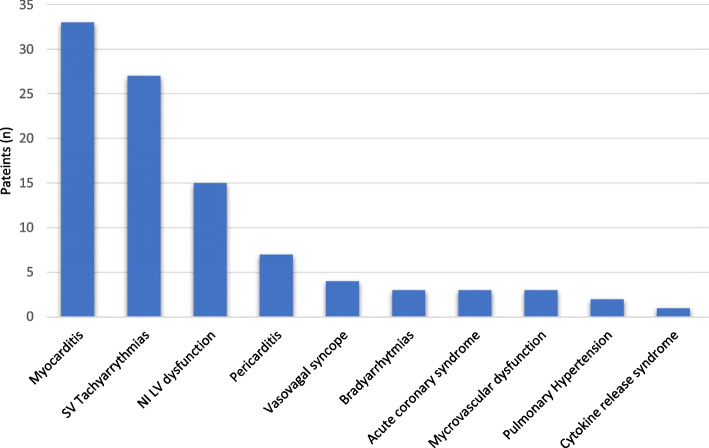
Incidence of cardiovascular adverse events in patients with immune checkpoint inhibitors. SV: supraventricular. NILV: non-inflammatory left ventricular

The total number of patients who presented with cardiovascular complications while being treated with ICI therapy was 89 (3.4%). Patients in this series presented with a broad spectrum of cardiovascular diseases judged to be related to their immunotherapy (Fig. 4). The median time from starting the immune checkpoint inhibitor to the development of any cardiovascular event was 14 weeks (IQR: 4.5–35). Patients receiving ICI therapy may develop more than one immune-related adverse event. In this cohort, 60% had an additional non-cardiovascular immune-related toxicity concomitantly or before the cardiac toxicity. Nine patients developed more than one ICI-related cardiovascular event (e.g., myocarditis and acute coronary syndrome).

#### Myocarditis

Myocarditis was the most common cardiovascular complication. Thirty-three patients were diagnosed with ICI-related myocarditis, of which 17 had “Definite Myocarditis,” 9 “Probable Myocarditis,” and 7 “Possible Myocarditis” according to Bonaca et al.’s definition criteria [3]. 73% (24/33) of the patients with myocarditis had a second immune-related adverse event such as colitis (10 patients), hepatitis (7 patients), thyroiditis (3 patients), myositis (2 patients), pneumonitis (1 patient), and hypopituitarism (1 patient). Ten out of these 24 patients presented with myocarditis and two or more immune related adverse events.

Treatment was implemented by the cardio-oncology team and the referring oncologist. Twenty-four patients (73%) received high-dose intravenous steroids, as first line treatment, following current recommendations [4]. High-dose steroid therapy was initiated as soon as the diagnosis was confirmed. In clinically unstable cases, the first dose of intravenous steroids was given prior to CMR or endomyocardial biopsy if clinical suspicion was high. Five, out of the 24 patients, had steroid-refractory ICI-related myocarditis and required a second immunosuppressant agent (mycophenolate mofetil). In three cases, myocarditis recurred after stopping IV steroids and needed a second course of this treatment in 2 patients and mycophenolate in the third case. Two patients received oral steroids when myocarditis was confirmed as they both had had a course of intravenous steroids to treat another severe non-cardiac immune toxicity. One patient presented with cytokine release syndrome requiring treatment with Tocilizumab, an interleukin six inhibitor, and three weeks later developed myocarditis, for which methyl-prednisolone was prescribed. Finally, 6 patients did not receive any active treatment at the instigation of our team, as the active inflammation had fully resolved by the time of referral, assessment and diagnosis. One of these patients, who had had a mild and uncomplicated myocarditis episode, successfully restarted a second phase ICI treatment without recurrence of myocarditis.

Using the management pathway described, with early use of high dose steroids, cardiac mortality rate was zero after a median follow-up time of 222 days (IQR: 57–705). All-cause mortality was 18% (6/33) being disease progression the cause of death in all cases.

#### Tachyarrhythmia

The second most frequent cardiac event was atrial tachyarrhythmia, with an incidence of 1% (27 patients). New atrial fibrillation (AF) was diagnosed in 13 patients, with ten being primary, 2 secondary to thyrotoxicosis, and 1 in the context of cytokine release syndrome. Ventricular arrhythmias (non-sustained ventricular tachycardias) were rare, diagnosed in 3 patients and always in the context of myocarditis.

Arrhythmias were treated as per current European Society of Cardiology guidelines [5–7].

Anticoagulation was started on 7 patients diagnosed with AF according to CHADS_2_VASC and HAS-BLED scores. In regard to rate and rhythm control; 5 primary AF cases were paroxysmal and did not require long-term rate or rhythm control treatment, 4 were persistent but asymptomatic and treated with rate control medication and only one case of primary AF case required ablation for refractory symptoms. Management of the cases of secondary AF was focussed on treating the underlying cause, although one patient with signs of acute heart failure required cardioversion with amiodarone. There were no deaths directly related to tachyarrhythmia.

#### Non-inflammatory left ventricular dysfunction

Fifteen patients (17%) developed new left ventricular dysfunction in the absence of myocarditis, ischaemia, infarction or other acute causes. This was classified as non-inflammatory left ventricular dysfunction. The ventricular impairment was diagnosed with echocardiography and/or cardiac magnetic resonance together with cardiac biomarkers and clinical findings. Cardiac magnetic resonance and troponin measurement were also used to rule out myocarditis or other differential diagnosis. Seven patients had a stress study − 3 stress echocardiograms and 4 perfusion CMR − 100% of which were negative for ischaemia. Thirteen patients had a CMR, late gadolinium enhancement was present in only 5 patients (38%) whereas T2STIRS and native myocardial T1 and T2 were normal in all these patients showing no evidence of myocardial oedema or active inflammation. One patient underwent an 18 FDG PET-CT which showed no abnormal myocardial uptake. As myocarditis had been excluded these patients were not treated with steroid therapy but with a combination of heart failure therapy and temporary interruption of the immune checkpoint inhibitor. 73% of the patients were started on angiotensine-converting enzyme inhibitors or angiotensine receptor blockers, 47% on betablockers, 13% on aldosterone receptor antagonists and 13% on Ivabradine. ICI treatment was restarted safely on 11 patients while switched to another therapy on 2 patients who had persistent LV dysfunction despite medical therapy. Information regarding medical therapy and continuity of ICI therapy was not available for 2 patients.

#### Differences between ICI-related myocarditis vs. non-inflammatory left ventricular dysfunction

Cancer patients who developed myocarditis did not differ from patients with NILVD in demographic variables such as age (median (IQR): 66 years (61–72) vs. 72 years (41–79), *p* = ns) and gender (61% male vs. 73%, *p* = ns). Most cardiovascular risk factors were evenly distributed among the two groups, although previous cardiovascular disease was more frequent in the NILVD group (9% vs. 40%, *p* = 0.018) while the rate of hypertension was significantly higher among patients who developed myocarditis (42% vs. 7%, *p* = 0.018). The most frequently used ICI in patients who developed myocarditis was combined therapy with Ipilimumab and Nivolumab (11/33 patients) whilst this therapy was not associated with NILVD. This difference was statistically significant (*p* = 0.011). Pembrolizumab usage was more frequent in the NILVD patients (8/15 vs. 7/33) although this difference was not significant (*p* = 0.09) and may reflect the indication for the drug and patients’ higher cardiovascular risk factor profile (lung and urological cancers). After conducting a multivariable analysis, hypertension and a second immune related adverse event were the only factors independently associated with a higher risk of developing myocarditis (*p* = 0.045, OR: 4.78 and *p* = 0.028, OR: 2.03 respectively).

**Fig. 5:**
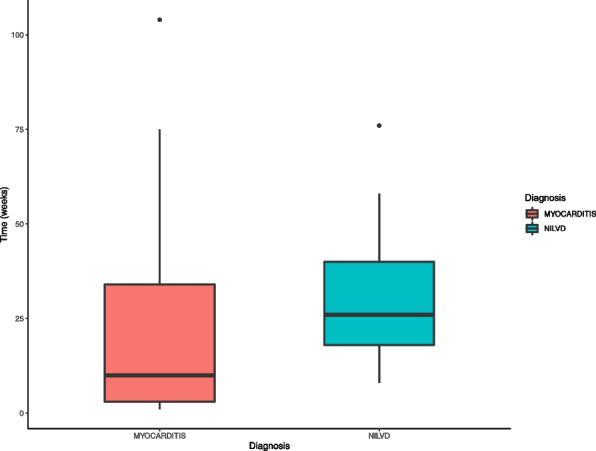
Time to diagnosis after first dose of the immune checkpoint inhibitor. The median time to diagnosis of Myocarditis was 12 weeks resulting significantly shorter than the time to diagnosis of non-inflammatory left ventricular dysfunction (26 weeks), *p* = 0.049

Several differences were identified between patients with myocarditis and NILVD, although some are self-fulfilling based on the definition criteria (Table 2). One main difference is the duration of ICI treatment prior to presentation with the cardiac event. The median ICI treatment time was 12 weeks (IQR: 3–34) in the myocarditis cohort versus 26 (IQR: 16.5–37) in the NILVD cohort (*p* = 0.049) (Fig. 5). Patients with myocarditis were more likely to have another immune-related toxicity at presentation (73% vs. 26%, *p* = 0.004). Patients with NILVD had reduced left ventricular systolic function compared to the myocarditis cohort. The median LVEF by echocardiography in the NILVD cohort was 49% (14.5), compared with a median ejection fraction of 60% in the myocarditis group (*p* < 0.001). Myocarditis patients had a higher incidence of late gadolinium enhancement in the left ventricular myocardium on CMR compared to the NILVD cohort (73% vs. 33%, *p* = 0.04), and a higher rate of active inflammation with T2STIR abnormal findings (27% vs. 0%). It is important to note that many patients had the CMR performed after treatment with high-dose steroids had been started, which may have influenced the relatively low incidence of positive T2STIR found in the myocarditis group compared with previously published findings [8]. The right ventricular ejection fraction, when assessed by CMR, was also significantly lower in patients with NILVD compared to ICI-related myocarditis (63% vs. 49%, *p* < 0.01), suggesting biventricular involvement. Myocarditis patients presented with a higher rate of troponin elevation (61% vs. 13%, *p* = 0.004), while there were no differences in proportion of elevated natriuretic peptides as they were frequently elevated in both cohorts (81% vs. 73%, *p* = 0.41). The two patients with elevated troponin in the NILVD had acute kidney disfunction with glomerular filtration rate < 20 ml/min/1.73². Biomarker elevation was stable and was considered to be related to renal failure rather than a sign of myocardial inflammation.

**Table 2:** Comparison between Myocarditis and Non-Inflammatory Left Ventricular Dysfunction

Characteristics	Myocarditis (33)	NI LV dysfunction (15)	*P*
**Age (median, IQR)**	66 (11)	72 (38)	0.61
**Male (n, %)**	20 (61)	11 (73)	0.52
**BMI (mean, SD)**	28 (5)	25 (5.6)	0.16
**Smoker (n, %)**	6 (18)	4 (27)	0.71
**Dyslipidaemia (n, %)**	10 (30)	6 (40)	0.51
**HTN (n, %)**	14 (42)	1 (7)	**0.018**
**DM (n, %)**	3 (9)	1 (7)	1
**Previous cardiac disease (n, %)**	3 (9)	6 (40)	**0.018**
**Previous treatment with anthracyclines**	3 (9)	1 (7)	**1**
**Troponin elevation (n, %)**	20 (61)	2 (13)	**0.004**
**Increased BNP (n, %)**	27 (81)	11 (73)	0.46
**Other Immune toxicity (n, %)**	24 (73)	4 (26)	**0.004**
**Echocardiogram**			
** LVEF (median, IQR)**	60 (9)	49 (14.5)	**< 0.001**
** LVEF < 50**	2 (6)	11 (73)	**< 0.001**
** CMR**	*n* = 33	*n* = 13	
** LVEF (median, IQR)**	65 (10)	51 (17)	**< 0.001**
** Positive LGE (n, %)**	24 (73)	5 (38)	**0.044**
** Positive T2STIR (n, %)**	9 (27)	0 (0)	**0.004**
** RVEF (median, IQR)**	63 (13)	49 (8)	**< 0.001**
** Other Immune toxicity (n, %)**	24 (73)	4 (26)	**0.003**
** Immune Checkpoint inhibitor**			
** Pembrolizumab (n, %)**	7 (21)	8 (53)	0.09
** Ipilimumab + Nivolumab (n, %)**	11 (33)	0 (0)	**0.01**
** Atezolizumab (n, %)**	3 (9)	2 (13)	1
** Avelumab (n, %)**	1 (3)	1 (6)	0.23
** Ipilimumab (n, %)**	2 (6)	1 (6)	0.16
** Nivolumab (n, %)**	2 (6)	2 (13)	0.69
** Durvalumab (n, %)**	5 (15)	0 (0)	0.16
** Dostarlizumab (n, %)**	1 (3)	0 (0)	1
** Spartalizumab (n, %)**	1 (3)	0 (0)	1

*HTN *Hypertension, *DM *Diabetes mellitus, *BNP *Brain natriuretic peptide, *LVEF *Left ventricular ejection fraction, *CMR *Cardiac magnetic resonance, *LGE *Late gadolinium enhancement, *RVEF *Right ventricle ejection fraction, *CVAE *Cardiovascular adverse event

#### Other cardiovascular toxicities

Other cardiovascular complications were pericarditis (7 patients), ischaemic heart disease (6 patients), vasovagal syncope (4 patients), bradyarrhythmia (3 patients) including one third degree atrio-ventricular block with pacemaker implantation, new pulmonary artery hypertension (2 patients) and cytokine release syndrome (1 patient) (Fig. 4). Three, out of the 6 patients with ischaemic heart disease, presented with unstable angina and were diagnosed with microvascular dysfunction through perfusion cardiac magnetic resonance and no significant coronary artery disease on the CT coronary angiogram. Two patients presented with NSTEMI, urgent angioplasty was performed in one case and medical treatment in the second one. Finally, there was one case of acute myocardial infarction who, by the time he was referred, Q waves had already developed. The patient underwent a CMR and CTCA, and a decision for medical treatment was made. The 2 cases with new pulmonary hypertension occurred in the absence of left ventricular dysfunction or pulmonary emboli and in temporal correlation to the use of immune check point inhibitors.

## Discussion

Immune checkpoint inhibitors have a clinically significant impact on cancer survival proven in randomised controlled trials and are licensed for the treatment of various cancers. This therapy has had a particularly dramatic effect on the outcomes for patients with cancers previously associated with high mortality, such as metastatic melanoma, metastatic renal carcinoma and advanced lung carcinoma [9]. Adjuvant and neoadjuvant indications are also now indicated for some cancers and are being assessed across a range of malignancies. Immunotoxicities are a common side effect of ICI, with as many as 70% of patients who may develop immune related adverse events and 40% of them needing to have their therapy interrupted [10, 11]. The importance of understanding the range of cardiovascular risks associated to the use of ICI therapies increases with the expanding number of licensed indications for this treatment, as does an understanding of the risk:benefit in specific cohorts of cancer patients where the absolute benefit is lower (adjuvant and neoadjuvant indications). In the last six years, the focus has been on myocarditis as the most serious cardiovascular adverse event caused by ICI therapy. This paper describes a widening spectrum of ICI-related cardiac complications including arrhythmias, NILVD, pericarditis, and acute coronary syndrome. While not intending to imply causation, given this is a descriptive study of our real-life experience and not a randomised control trial, we believe the reported findings are of high relevance to the cardio-oncology community.

Myocarditis was the most frequent ICI-related cardiovascular complication in our cohort. There were rare case reports prior to 2016, but awareness increased after Johnson et al. reported two cases of fulminant myocarditis with the use of combined therapy, Ipilimumab and Nivolumab. The authors reported that myocarditis was a rare complication (0.09% cases treated with ICI) but with high mortality (33%) [11]. Since this first publication, further cohorts have been published with increasing numbers of patients and events [4, 12–15]. Diagnostic criteria have evolved and in the latest definition from the International Cardio-oncology Society, myocarditis is confirmed either by histopathology (endomyocardial biopsy) or the combination of a new cardiac troponin rise and either a diagnostic CMR or at least 2 minor criteria [16]. In this cohort, among the 17 patients characterised as definite myocarditis, 11 (65%) fulfilled this new definition. Four patients (44%) with probable myocarditis met the criteria as well as 3 out of the 7 patients with possible myocarditis. Non-invasive imaging techniques (echocardiography, CMR), ECG, ECG telemetry and cardiac biomarker measurement are recognised as critical investigations [17] in this context and should be performed rapidly in cancer patients receiving ICI therapy who have suspected myocarditis [3]. In the cohort presented in this article the use of all these diagnostic tools, together with prompt treatment with high dose steroids at first instance, led to outstanding clinical outcomes with no cardiovascular deaths after > 200 days of median follow-up time.

The question of routine surveillance for cardiovascular complications and its utility in patients under ICI treatment has been raised before [18]. This is a cohort of patients which includes cases as early as from 2014 when no formal recommendations were available. Cardiovascular investigations were mainly driven by signs or symptoms of cardiac disease.

and decided by the treating team. Our current recommendation is to follow the recently published cardio-oncology guidelines [19] where ECG, NP and cTn measurements are recommended in all patients before starting ICI therapy and before doses 2, 3 and 4 to detect subclinical ICI-related CV toxicity.

In clinical practice some cases of myocarditis are more challenging to diagnose. One example is that of the cancer patients who have received steroid treatment for another immune related toxicity, with myocarditis suspected subsequently. Partially treated ICI-related myocarditis may not meet the formal criteria, e.g. CMR evidence of prior myocarditis on late gadolinium enhancement but without active inflammation. In cases where diagnostic uncertainty exists, we recommend cardiac PET-CT for clinically stable patients, as suggested by Boughdad et al. who demonstrated that this method is highly sensitive in detecting myocardial inflammation [20], although further research is still needed. In cases of clinical instability and uncertain diagnosis, endomyocardial biopsy is the diagnostic tool of choice.

The frequency of ICI-related myocarditis in this study, is substantially higher than initially recognised, when the reported rate was 0.09%, and more similar to, although slightly higher than, that reported in 2018 by Mahmood et al., 1.14% [21]. Our study presents a cancer population treated with ICI therapy of 2647 patients with a rate of myocarditis of 1.25% (33/2647). In this cohort, there were no cardiovascular deaths reported among the patients with ICI-related myocarditis. This is in stark contrast to the 17–27% mortality in previously published cohorts [21, 22] and there are several factors which we believe may explain the difference. Firstly, the mentioned publications included patients until 2017. Our study has included cases until December 2020 and 56% of the patients had been referred in the last 2 years. This is very likely to have had an impact on the outcomes since, over several years, both oncologists and cardiologists in the two centres have developed a high index of suspicion to consider the possibility of, and investigate for, myocarditis. This has led to a reduction in the threshold for referral of cancer patients with suspected ICI-related myocarditis by the oncologists and rapid assessment by the cardio-oncology service, with the consequence of early diagnosis and treatment. Most patients diagnosed with myocarditis, or at high clinical suspicion and where clinically unstable, were started on high-dose steroids and ICI treatment interrupted. The current protocol used in our service is the following: intravenous methylprednisolone 500–1000 mg once daily for three days minimum and continuing until troponin stabilises at < 80 ng/L and any clinical complications (heart failure, ventricular arrhythmias) have settled, then switching to oral prednisolone 1 mg/kg with weaning scheme whilst monitoring cardiac troponin. Early treatment of ICI-myocarditis with high dose steroids is believed to reduce MACE and cardiovascular mortality [23], and this management pathway is probably another reason for such low mortality rates.

This paper also raises awareness that there are other ICI-related cardiovascular complications besides myocarditis. Tachyarrhythmias, such as atrial fibrillation, were among the most frequent. Pericarditis is also observed, either with myocarditis (peri-myocarditis), or as a separate diagnosis. Less frequent, but of high clinical importance is ICI-related ischaemic heart disease, including acute myocardial infarction with the need of urgent percutaneous coronary intervention; bradyarrhythmia leading to pacemaker implantation; and pulmonary artery hypertension.

ICI treatment is usually interrupted in cancer patients who develop ICI-related cardiovascular complications until the diagnosis is established. This is critical as myocarditis is a relative contraindication to further ICI treatment. In contrast, it may be restarted in patients with other ICI-related cardiac events, including AF, pericarditis and NILVD, after this has been treated. The current approach is to follow general cardiology guidelines for these other ICI-related cardiac toxicities, except that ICI-related pericarditis may require steroid therapy in addition to colchicine.

One of the most important aspects of our study is the description of NILVD as a new cardiovascular complication associated with the use of immune check-point inhibitors. This event was observed in 0.6% of the referred patients (15/2647) and happened in those who remained on ICI treatment for longer courses, the median time to presentation being 26 weeks after starting therapy. Several other characteristics distinguish myocarditis from NILVD including the increase in cardiac troponin and the presence of late gadolinium enhancement on CMR, which are both higher in myocarditis, given the inflammatory nature of this complication, and the requirement of these tests to be abnormal for the confirmation of the diagnosis of ICI-related myocarditis. Left ventricular systolic function was lower in the NILVD cohort. We also observed that concomitant right ventricular dysfunction was a frequent finding in these patients, suggesting that longer-term ICI treatment may lead to myocardial impairment in a subgroup of patients, affecting both ventricles, via a non-inflammatory mechanism. We have initiated guideline-based heart failure treatment, temporarily interrupted ICI treatment in severe cases, and then restarted ICI treatment after recovery of ventricular function. On the other hand, LV dysfunction was only present in 27% of myocarditis patients, stressing the point that myocarditis should not be ruled out on the basis of a normal LV function, this is aligned with previously published observations [21]. Finally, several risk factors were associated with myocarditis but not NILVD such as the presence of other immune related toxicity and the use of combined therapy (Ipilimumab and Nivolumab). Therefore, it appears that immune checkpoint inhibitors can trigger two different types of myocardial injury: myocarditis via active inflammation with T lymphocyte infiltration of the myocardium and a non-inflammatory type of ventricular dysfunction. The pathological mechanisms leading to NILVD are unknown, although in the year 2001 Nishimura et al. showed that PD1 knock-out mice develop a form of dilated myocardiopathy, implying that this receptor is crucial to maintaining myocardial vitality and blocking it might lead to myocardial dysfunction [24] Further research in the field is required to better understand the pathophysiology, diagnosis, and treatment of both ICI-related myocarditis and ICI-related NILVD.

### Limitations

There are several limitations to this study that the authors would like to acknowledge. First, this is a single-centre study limiting the cases to a relatively low number compared to previous pharmacovigilance publications [2]. However, we believe that data proceeding from real world experience during routine clinical practice are increasingly valuable as a resource and significantly complement the information coming from these other sources. Secondly, the patients included were referred by the oncology and haematology teams which might have led to referral bias. There might be other cases which were not referred due to non-cardiac causes and therefore not included, or cases in which cardiovascular complications may not have been recognised or considered to be associated with ICI therapy. This limits the possible conclusions regarding the incidence of cardiovascular events in the wider population of patients treated with ICI. Furthermore, a matched untreated control cohort was not available to compare the cardiac event rates spontaneously occurring in this group of patients and therefore conclusions regarding causality cannot be drawn and the number of endomyocardial biopsies was limited to 2, not allowing to perform any statistically valid comparison between the histological and clinical findings. Finally, specific data points, such as the date of ICI treatment commencement or the number of cycles received prior to developing the adverse events, were not available in all cases.

## Conclusion

There is a spectrum of cardiovascular complications observed in cancer patients treated with ICI, which is broader than initially described. Myocarditis, NILVD, arrhythmia, ischaemic heart disease and new pulmonary artery hypertension were observed during treatment with immune checkpoint inhibitors. ICI-related myocarditis remains the most frequent cardiovascular event, with an incidence of 1.24%. It usually presents in the first four cycles of ICI treatment and is associated with other immune-related toxicities and dual ICI therapy. We describe ICI-related NILVD, a new entity observed in patients on long-term immunotherapy, requiring heart failure medication and temporary ICI interruption, but no steroid therapy. The overall percentage of patients who developed cardiovascular events in this cohort was 3.4%. We believe that this is likely to increase as the number of licensed indications for immune checkpoint inhibitors expands, cardiologists and oncologists become more aware of the broad spectrum of cardiac events and more cancer patients survive and receive longer ICI treatment courses.

## Data Availability

The dataset used for this study, with the anonymised patients’ data, is held by the authors and would be available for review upon request.

## Abbreviations

ICI: Immune checkpoint inhibitors
ECG: Electrocardiogram
CMR: Cardiac magnetic resonance
PET-CT: Positron emission tomography-computed tomography
cvAEs: Cardiovascular adverse events
NILVD: Non inflammatory left ventricular dysfunction
IQR: Interquartile range
AF: Atrial fibrillation
LVEF: Left ventricular ejection fraction

## Declarations

The remaining authors have nothing to disclose.
